# Correland^SW^: Correlation Networks from LC–MS Data

**DOI:** 10.1021/jasms.6c00065

**Published:** 2026-04-22

**Authors:** Andrea Kosinova, Jiri Gruz

**Affiliations:** Department of Experimental Biology, 48207 Palacky University, Slechtitelu 27, 78371 Olomouc, Czech Republic

**Keywords:** metabolomics, correlation, network, LC-MS data

## Abstract

The increasing size and complexity of mass spectrometry (MS) data sets necessitate advanced computational tools. This study presents Correland, a MATLAB-based software for clustering and visualizing metabolite correlations through weighted correlation networks, which directly represent pairwise associations. Its effectiveness was tested on a data set from nontargeted LC-MS analysis of 14-day-old *Arabidopsis thaliana* seedlings inoculated with *Alternaria alternata* and *Fusarium oxysporum*, demonstrating effective clustering of biosynthetically related metabolites. The ion grouping algorithm resulted in a substantial reduction in network scale (83 nodes/metabolites from approximately 900 features). In addition to network construction, Correland enables pseudomolecular ion identification with a success rate of 86–90% achieved in *Arabidopsis* extract. Network density is reduced by limiting visible edges, producing interpretable and visually coherent networks generated in a single step using rescaled Pearson correlation coefficients.

## Graphical abstract

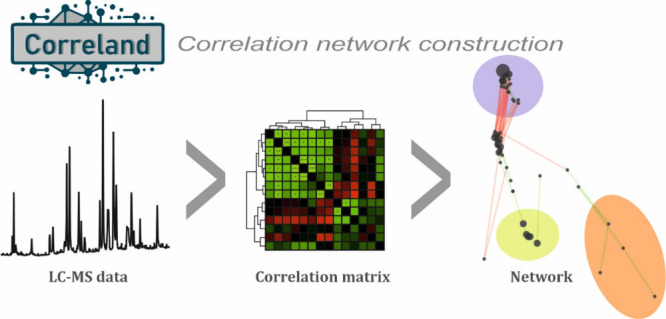

## Introduction

Metabolomic analysis represents a widely used approach for the systematic exploration, identification and quantification of metabolites in a biological sample. A nontargeted analysis, which refers to the analysis of all metabolites, is frequently performed using liquid chromatography–mass spectrometry (LC–MS). This analytical technique generates thousands of features (peaks) characterized by their *m*/*z*, retention time (RT), and peak area. To reveal patterns and reduce the complexity of such high-dimensional data sets, multivariate statistical approaches such as hierarchical clustering analysis (HCA) and principal component analysis (PCA) are commonly applied, although they have inherent limitations in representing complex relationships.^1^ HCA provides a tree-like structure that emphasizes similarity within clusters but often obscures cross-cluster associations. PCA reduces dimensionality effectively, yet the resulting components may be difficult to interpret biologically and may overlook nonlinear dependencies. In contrast, correlation-based network visualizations allow metabolites to be represented as interconnected nodes, directly capturing the strength and direction of pairwise associations. This approach highlights not only groups of highly correlated metabolites but also bridging features that connect different metabolic pathways, thereby offering a more intuitive and biologically meaningful representation of the underlying metabolic landscape. The use of network visualization is becoming increasingly prevalent in a number of academic disciplines, including the social sciences, ecology, neuroscience and genetics.^2^ The process of visualization of network clusters is generally described as a spatialization, which refers to the process of assigning positions to nodes in a network using layout algorithms.^2^ Correlation network based on force-directed layout is used to graphically visualize the attractive forces between adjacent nodes and repulsive forces between distant nodes. If used with LC-MS data, the nodes represent ions/metabolites, while the edges represent correlation between them. Network diagrams are seen as a valuable tool for gaining insights, although there are also concerns that they can be misinterpreted.^2^ The clarity of networks is frequently ensured by the restriction to a small number of nodes or reduction of the number of edges.^3^ Therefore, the majority of software tools developed are focused on the creation of small networks of adducts and fragments originating from a single metabolite, coupled with the annotation of these networks using diverse metabolic libraries. However, the construction of correlation networks derived from a large number of metabolomic data has the potential to reveal the interesting relationships among metabolites, which can subsequently be subjected to further investigation.

## Materials and Methods

### Software Requirements

Correland is a standalone MATLAB-based application. The installer includes the necessary MATLAB Runtime R2019a and requires administrator rights on Windows. Installation files and video tutorials are available at https://www.prf.upol.cz/keb/correland/.

### LC–MS

The Correland was thoroughly tested using a data set of 12 plant samples analyzed in positive and negative ion modes (data are included in the installation package as an Excel file). The samples analyzed were 14-day-old seedlings of the model plant *Arabidopsis thaliana*, which had been inoculated with two fungal strains, namely *Alternaria alternata* and *Fusarium oxysporum*. All variants consisted from 4 replicates. Briefly, 100 mg of plant material was homogenized and extracted in 1 mL of 80% methanol using an oscillation ball mill. The extract was evaporated and redissolved in 20% methanol before LC–MS analysis. The LC-MS system consisted of UHPLC coupled to QTOF-MS (Synapt G2-Si, Waters). The analytes were ionized using an electrospray ionization (ESI) source (capillary voltage 2.5 kV, cone voltage 25 V) operated in both positive and negative ionization modes. The Excel list of features was generated by in-house metabolomic MATLAB algorithms. The LC–MS conditions and data processing workflow are described in detail by Rarova.^4^

## Results

### Software Description and Architecture

Correland is a software tool designed with the specific purpose of spatializing metabolites and their relationships in a correlation network. The pairwise correlations are calculated from individual concentration patterns. The summary of the overall Correland procedure and related input parameters are summarized in a Figure 1.

**Figure 1:**
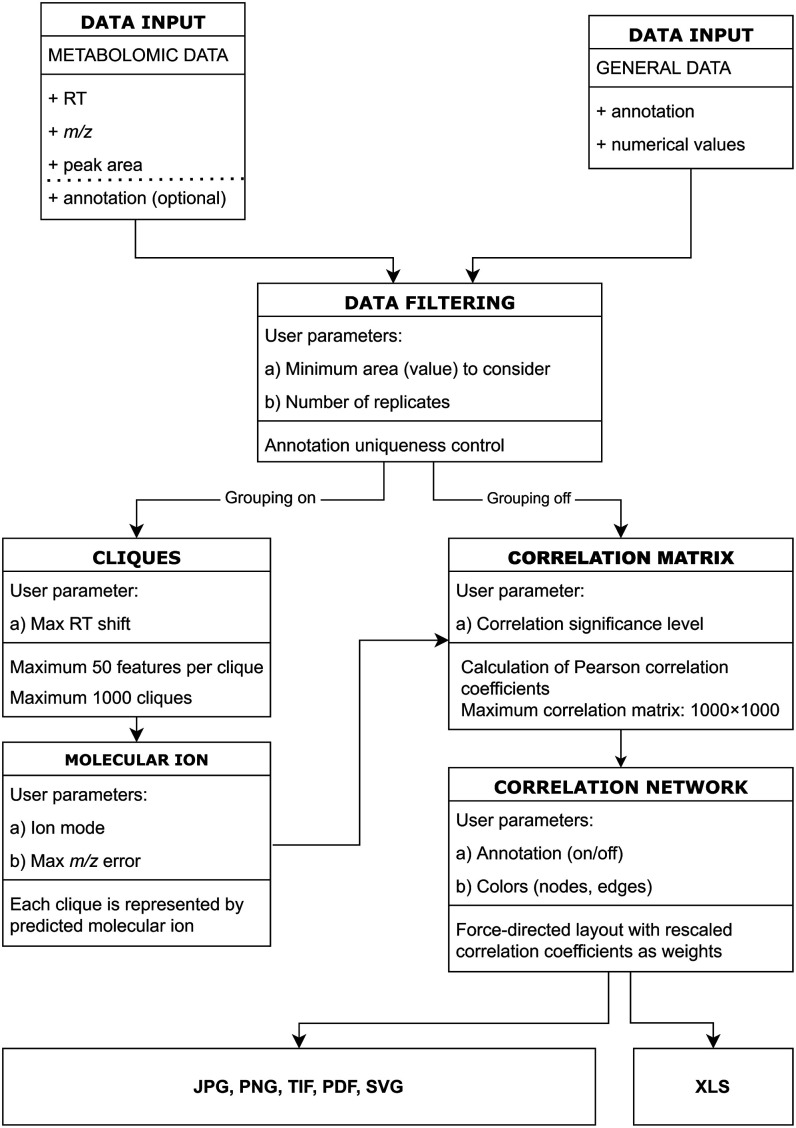
Correland workflow scheme.

### Data Structure

The LC–MS data can be imported to Correland as an Excel file with a specific structure, where each row contains a unique feature/metabolite characterized by mass-to-charge ratio (*m*/*z*), retention time (RT), peak area and text annotation (optional). The typical excel data set is included in the installation package for testing. Data can be preprocessed (e.g., log-transformed) by the user before loading it into Correland based on the experimental context and goals; removal of outliers prior to analysis is highly recommended.

### Ion Grouping

To avoid excessively long calculation time, ions originating from a single metabolite can be grouped to form a single clique (group), which is further represented by the pseudomolecular ion (section titled Pseudomolecular ion identification). The related ions are identified based on the similarity of their retention times and stable ratio of their intensities among samples. Based on these assumptions, the clique is identified as follows: the feature with the largest area (hereafter referred to as the “base peak”) from the list of features is grouped with all features that have (i) the same RT (±RT shift) and (ii) the same ratio to the base peak (RSD < 25%) in all samples. The resulting cluster (clique) is removed from the list and the procedure is repeated as many times as there are features left in the list.

### Pseudomolecular Ion Identification

The identification of deprotonated or protonated molecules (pseudomolecular ions) is based on a system of eqs (Figure 2) describing the *m*/*z* differences among all features originating from a single metabolite (clique). Correland includes a standard set of equations for typical LC–MS adduct and fragment ions, defined separately for positive and negative ionization modes. Each *m*/*z* difference related to expected adducts and fragments has a weight which is proportional to its relative importance in the pseudomolecular ion determination process.

**Figure 2:**
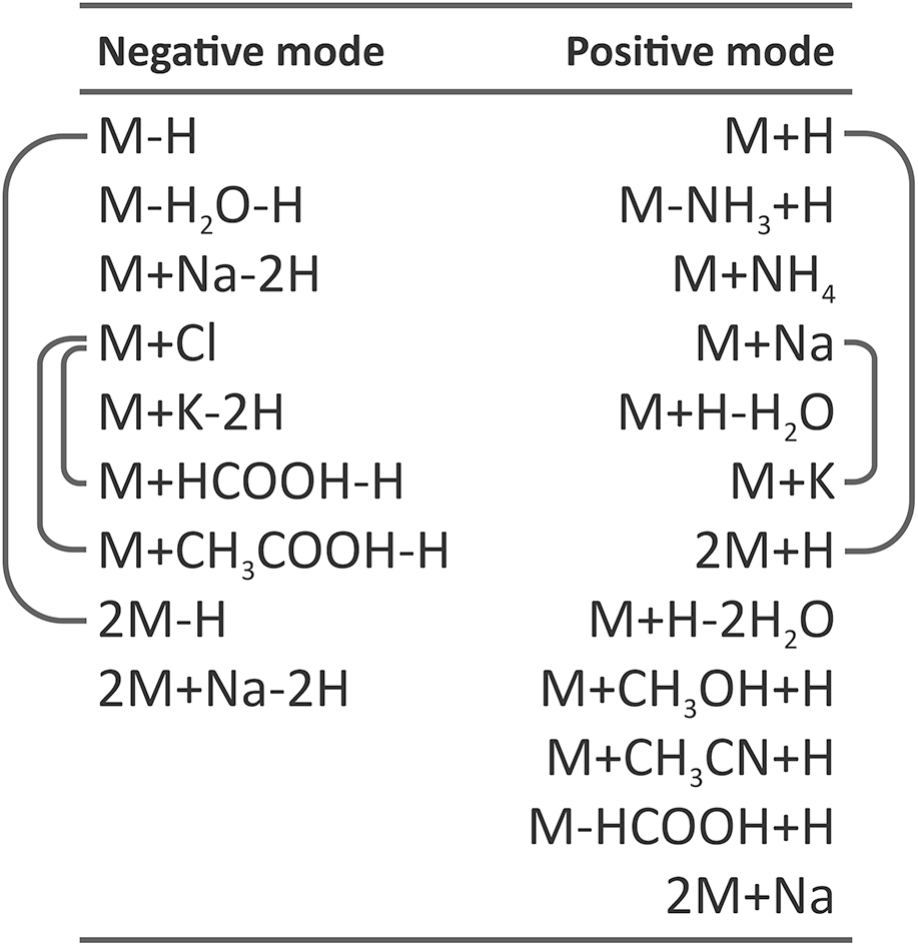
System of equations used for identification of pseudomolecular ions in negative (left) and positive (right) modes. The pairs of ions connected by line receive the highest predefined weight (*w*_r_), e.g., if ions potentially representing [M-H]^−^ and [2M-H]^−^ are both present in a clique, their *w*_r_ is set to 10, which typically results in the identification of pseudomolecular ions.

If the clique contains only a single feature (ion), it will always be selected as the pseudomolecular ion. Otherwise, the identification of the pseudomolecular ion is subject to the following steps:

All features are sorted by *m*/*z* in descending order. Each subcluster of features characterized by a maximum *m*/*z* difference of 1.008+er Da (er is the *m*/*z* measurement error) between all adjacent features is substituted/represented by the most abundant feature from the subcluster. In this way, the majority of isotopic peaks and coeluted compounds (Δ*m*/*z* < 1 Da) are excluded from the clique and subsequent calculations of the pseudomolecular ion.
The Δ *m*/*z* value of each pair of features from the clique is compared to the list of adducts and fragments, where each *m*/*z* difference (±er) has a predefined weight ranging from 0 to 10. The total weight of a matching Δ *m*/*z* is calculated as *w*_r_+ *w*_a_, where *w*_r_ is the predefined weight for the particular Δ *m*/*z* and *w*_a_ is the sum of both feature areas normalized to the base peak area. The suggested pseudomolecular ion with the highest sum of weights (total weight) is considered identified if the total weight is ≥4. In such case, no further steps are taken (see “The mass differences matching” in Supporting Information, that provide further insight into the weight settings). If the total weight is less than 4, the pseudomolecular ion is only putatively identified as a candidate #1 for further evaluation. There are additional criteria that must be met for candidate #1:
  a. The *m*/*z* value of calculated pseudomolecular ion is higher than or equal to the *m*/*z* value of the base peak.
  b. At least one Δ *m*/*z* matches that from the list of adducts and fragments.
  c. The calculated pseudomolecular ion is present within the clique, i.e., it was detected by LC-MS.
  d. If the above conditions are not met, there is no candidate #1.
A pseudomolecular ion candidate #2 is predicted by searching the clique for neutral losses, regardless of ionization polarity, that are characteristic for the analyzed sample, e.g. plant, fungi or animal. These losses are usually larger (e.g., sugar moiety), and they are assumed to result in the formation of the base peak which is a stable fragment. This simplification is based on the assumption that the presence of a high intensity adduct (base peak) would lead to the identification of the pseudomolecular ion already in step II (total weight ≥ 4). Therefore, only features with *m*/*z* values exceeding those of the base peak will be used to calculate the Δ *m*/*z*.
  a. The *m*/*z* differences between the base peak and the features from the clique are compared with the characteristic neutral losses from the list. If there are one or more matches, the feature with the highest *m*/*z* becomes candidate #2.
  b. The base peak (ion) is supposed to be candidate #2 if its *m*/*z* is the highest within the clique.
  c. If neither of the above conditions are met, there is no candidate #2.
If candidates #1 and #2 are the same, the pseudomolecular ion is identified based on this prediction and no further step is taken.
Candidate #3 is selected by the relative abundance of the detected features. Only features from the clique with area larger than 50% of the base peak and *m*/*z* higher than that of the base peak are considered. If such features are present, the one with the highest *m*/*z* is selected as candidate #3. Otherwise, the base peak becomes candidate #3.
If there are at least two existing and identical candidates out of the three suggested (#1, #2 and #3), the pseudomolecular ion is identified based on this prediction. Otherwise, the feature with the highest *m*/*z* from the candidates is identified as a pseudomolecular ion.

### Correlation Network

The basis for the generation of the correlation network is the correlation matrix, where each pair of features is characterized by a Pearson correlation coefficient *r* and a *p*-value. The significance level α can be set by the user, using either the Bonferroni correction or an unadjusted value, which may be more suitable for exploratory purposes and large LC–MS data. A low α value may result in a reduction in the number of nodes, as only features/metabolites with at least one significant correlation are included in the network.

The direct use of the Pearson correlation coefficient *r* as a weight in the force-directed layout results in insufficient spatial distribution and difficult interpretation of the network. In support of the distinction between positive and negative correlations, and to enhance the visual clarity of the network, *r* values are rescaled according to the following procedure:

For positive correlations *r* > 0, the weight is calculated as follows:

$$s(1-|r^{x}|)$$

For negative correlations *r* ≤ 0, the weight is calculated as follows:

$$s+|r^{x}|(1-s)$$

The parameter *s* denotes the central scaling value that separates positive and negative correlations in the rescaled space, while exponentiating correlation coefficients by *x* highlights small differences among values close to either 1 or −1, thereby enhancing the interpretability of strongly correlated variables. After rescaling, a positive correlation produces weights ranging from 0 to *s*, while negative correlation results to weights ranging from *s* to 1. By this way, negatively correlated nodes are pushed further from each other, while positively correlated nodes are pulled together, thus creating relatively small and well-defined clusters. There are three predefined combinations of *s* and *x* values that result in weak (*s* = 0.5 and *x* = 16), normal (*s* = 0.3 and *x* = 8) or strong (*s* = 0.1 and *x* = 2) clustering of nodes. The predefined values were tested with typical LC-MS data and optimized to support interpretable and visually clean graphical output. Network layout is generated using a force-directed MATLAB algorithm with the number of iterations scaled to the number of displayed nodes, resulting in an organic appearance with slight variability between runs. Network clarity is improved by displaying only the strongest correlation (edge) per node; additionally, one-third of the strongest correlations in the opposite direction are shown if their *p*-values pass the significance level α. This step yields a more balanced network in terms of positive and negative correlations and improves the overall layout with a typical node/edge ratio slightly above 1. The spatial distribution of the nodes remains entirely unaffected by this process.

There are several graphical outputs available, including an image, document and metafile (.png, .pdf, .tif and.svg).

### Illustrative Examples

The resulting correlation networks showing metabolites from *Arabidopsis thaliana* grown under biotic stress conditions are presented in Figures 3 and 4. Both networks were generated from the same datafile (included in the installation package) using identical user-defined parameters, with the only difference being the clustering intensity parameter (normal vs strong).

**Figure 3:**
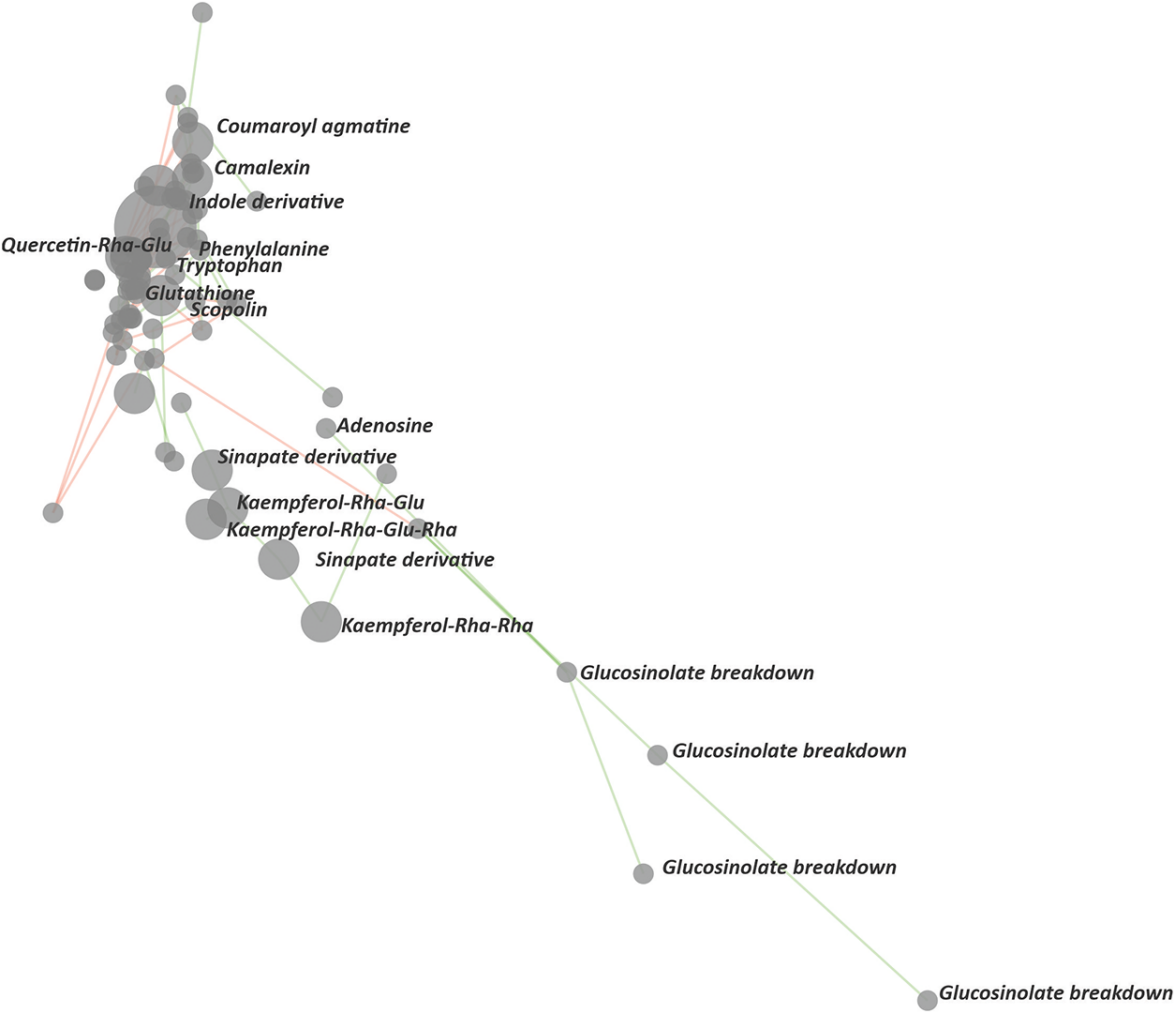
Normal clustering. The correlation network (83 displayed nodes, 10 iterations) for metabolomic data obtained by LC-MS analysis of 12 samples of *A. thaliana* seedlings inoculated with *A. alternata* and *F. oxysporum*. Green and red edges represent positive and negative correlations, respectively. Applied parameters: ion mode: positive; minimum area to consider: 9000; correlation significance level (α): 0.01 (unadjusted); Bonferroni correction: off; clustering: normal; ion grouping: on; max RT shift (min) 0.02; max *m*/*z* error (Da) 0.016. Spatialization quality metrics: mean Silhouette score: 0.628, number of clusters (DBSCAN): 2. The putative identification/annotation of metabolites was based on the comparison with local and public databases (NIST, MoNA, and KEGG).

**Figure 4:**
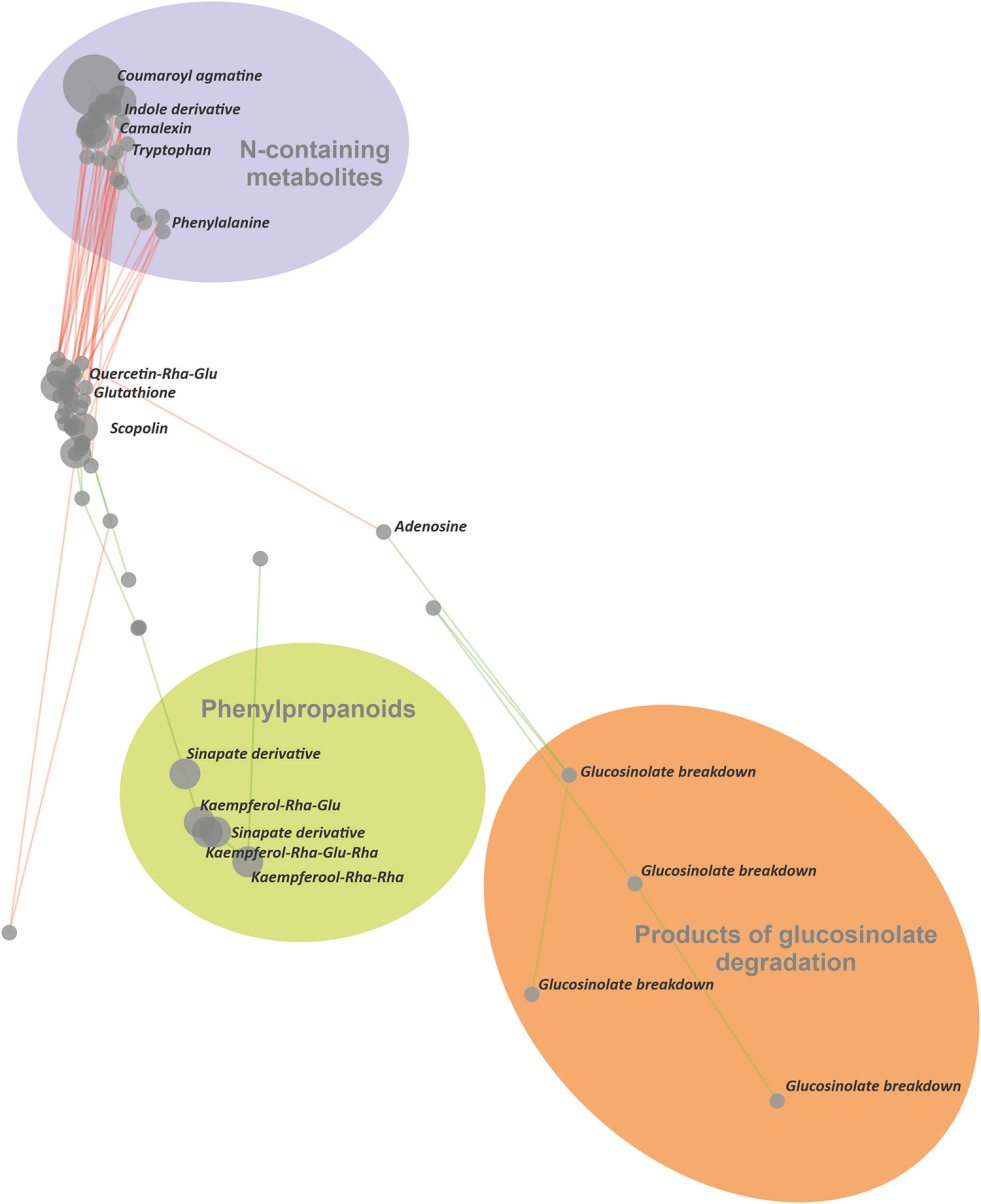
Strong clustering. The correlation network (83 displayed nodes, 10 iterations) for metabolomic data obtained by LC-MS analysis of 12 samples of *A. thaliana* seedlings inoculated with *A. alternata* and *F. oxysporum*. Green and red edges represent positive and negative correlations, respectively. Applied parameters: ion mode: positive; minimum area to consider: 9000; correlation significance level (α): 0.01 (unadjusted); Bonferroni correction: off; clustering: normal; ion grouping: on; max RT shift (min) 0.02; max *m*/*z* error (Da) 0.016. Spatialization quality metrics: mean Silhouette score: 0.779, number of clusters (DBSCAN): 4. The putative identification/annotation of metabolites was based on the comparison with local and public databases (NIST, MoNA, and KEGG). Group highlighting (ellipses) was applied following export from the Correland software.

In this example, normal clustering (Figure 3) results in only partial separation of functional clusters, whereas strong clustering (Figure 4) yields separation of metabolites that is both visually and biologically meaningful. This was supported by an increase in both the number of detected clusters and the Mean Silhouette Score, and further confirmed by preliminary identification and annotation of the displayed metabolites, demonstrating that the resulting clusters are functionally and/or structurally related. For instance, metabolites in the purple cluster showed strong accumulation under biotic stress, following the accumulation pattern of the defense metabolite camalexin.^5^ These metabolites predominantly contained nitrogen, indicating additional structural similarity. In contrast, the yellow cluster mainly comprised phenylpropanoids (flavonoids and cinnamates) that were generally depleted during stress experiments. Another clearly separated cluster contained products of glucosinolate degradation, including various isothiocyanates and nitriles.^6^ Overall, the method translates distinct accumulation patterns into a spatial organization of functionally related metabolites, thereby facilitating biologically driven interpretation and supporting hypothesis generation for subsequent experimental validation. Further application to the LC-MS metabolomic data describing an aging (3–92 weeks) mouse brain can be found in Figure S11—Supporting Information.^7^

The impact of ion grouping on network complexity is demonstrated in Figure 5, which shows a clique represented by sinapoyl malate, a major secondary metabolite from *Arabidopsis thaliana*. The efficiency of pseudomolecular ion identification was tested on a data set derived from the LC-MS analysis of *Arabidopsis thaliana*.

**Figure 5:**
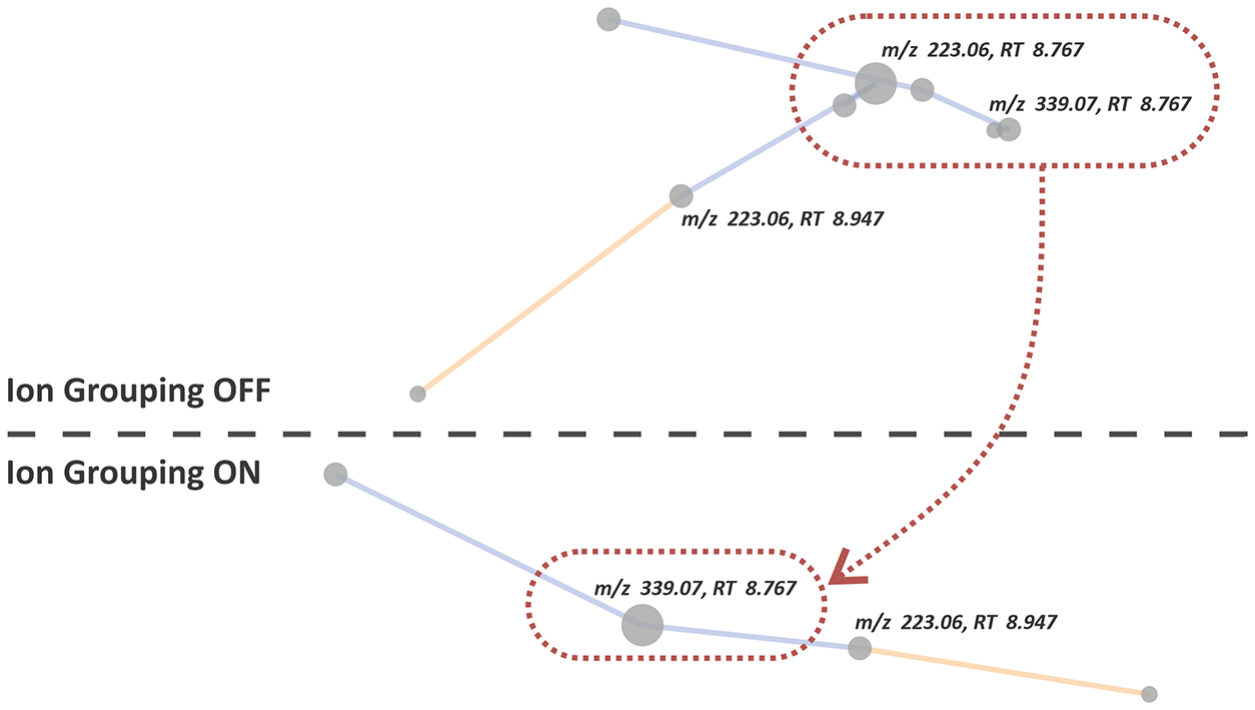
Comparison of a part of the correlation network before and after ion grouping. The red circle in the upper part (ion grouping OFF) shows a cluster of features derived from a single metabolite. This clique is represented by a single pseudomolecular ion of sinapoyl malate (*m*/*z* 339) after grouping and pseudomolecular ion identification (ion grouping ON). The resulting node size is proportional to the area of the base peak. Blue and orange edges represent positive and negative correlations, respectively.

To demonstrate the added value of Correland’s integrated workflow compared to a standard network construction approach, Figure 6 compares correlation networks built from the same data set using different processing strategies. Panel A shows the network exported from Correland, incorporating ion grouping, data filtering, significance testing, and Pearson correlation rescaling, resulting in a well-structured network with clearly distinguishable clusters (cf. Figure 4). Panel B presents a network generated in Cytoscape using the same Correland-preprocessed data but with standard Pearson correlation coefficients instead of the rescaled metric, yielding two visible clusters but with notably less spatial separation. Panel C displays a Cytoscape network built from unprocessed data using only Pearson correlation coefficients, which produced a single compact cluster with no apparent substructure.

**Figure 6:**
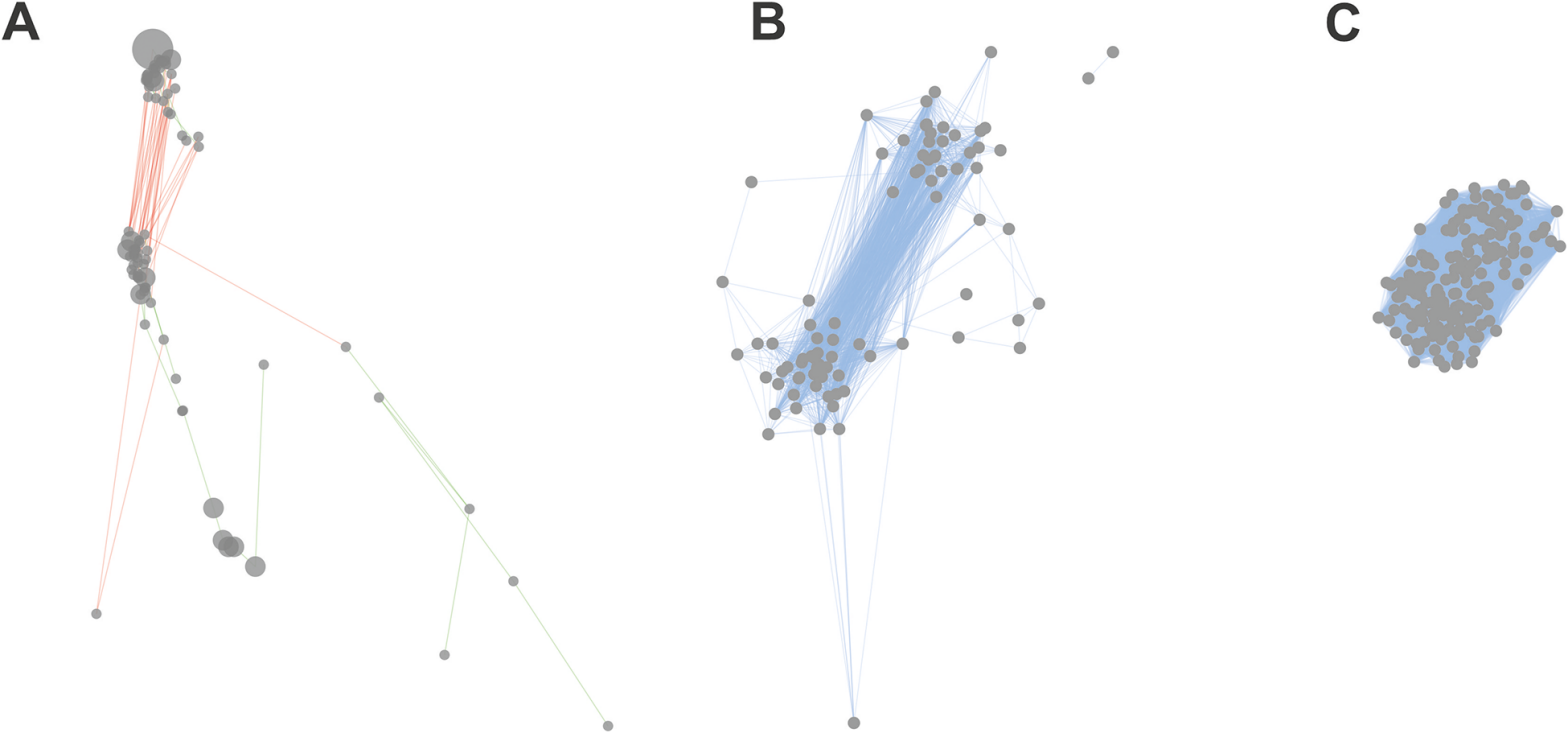
Comparison of clustering efficiency on metabolomic data obtained by LC–MS analysis of 12 samples of *A. thaliana* seedlings inoculated with *A. alternata* and *F. oxysporum* (data provided within the software). (A) Correland—ion mode: positive; minimum area to consider: 9000; correlation significance level (α): 0.01 (unadjusted); Bonferroni correction: off; clustering: normal; ion grouping: on; max RT shift (min) 0.02; max *m*/*z* error (Da) 0.016. (B) Cytoscape—prefuse force directed layout with Pearson correlation coefficient as edge weight; data were processed exactly as for Correland—the only exception is the direct use of Pearson correlation coefficient instead of rescaled metrics. (C) Cytoscape—prefuse force directed layout with Pearson correlation coefficient as edge weight; no preprocessing/ion grouping applied; calculated Pearson correlation coefficients were directly applied.

## Discussion

Generating a complete correlation network displaying all metabolites is a challenging task due to visual and computational limitations arising from the high number of nodes and edges.^8^ One of the widely used general-purpose network visualization tools is Cytoscape, which can also be employed to construct correlation networks; however, the user must supply a correlation matrix, which—depending on data preprocessing—may affect the resulting network (Figure 6). In particular, the absence of preprocessing collapsed the network into a single indistinct cluster (Figure 6C), highlighting the importance of integrated data curation for meaningful network spatialization. To the best of our knowledge, no standalone software currently combines a single step construction of concentration-based correlation networks and effective pseudomolecular ion identification. The majority of metabolomic tools are highly specialized and commonly implemented as Cytoscape plug-ins.^9^ For example, MetaNetter and MetaNetter2 focus on adduct network generation and visualization of adduct and transformation patterns.^10^ MetScape enables construction of metabolite correlation networks when pairwise correlation coefficients are provided, typically calculated using the Debiased Sparse Partial Correlation (DSPC) algorithm with multiple-testing correction.^11,12^ A more recent development, the R package DNEA,^13^ extends this concept by introducing data-driven feature grouping prior to network construction, although it may require user input or external databases. Both DSPC and DNEA rely on multistep workflows with external visualization tools, such as Cytoscape or the igraph R package (DNEA). Beyond Cytoscape, CAMERA is a Bioconductor package for adduct and fragment annotation linked to metabolite databases such as HMDB and UniCarb-DB.^14^ In contrast, Correland is a fully standalone application that enables quick generation of correlation networks across all detected metabolomic features using only *m*/*z*, retention time, and feature area, reflecting the increasing demand for database-independent visualization tools.^8^ The emphasis on simplicity and a low entry barrier for users also underpins several methodological choices for network construction in Correland. Pearson’s correlation coefficient was chosen as the default measure, as rank-based alternatives (Spearman, Kendall) tend to inflate correlations among low-variance background metabolites at small sample sizes and do not discriminate between large and trivial coordinated concentration changes. Partial correlation networks (e.g., Gaussian Graphical Models) offer a complementary approach by isolating direct metabolite associations; however, they require regularization in the typical metabolomics scenario where *n* ≪ features, introduce additional parameter choices, and yield sparser networks that may not capture the broader coregulation patterns.

Accurate identification of pseudomolecular ions is a key step in metabolite annotation and data dimensionality reduction.^15^ CliqueMS approaches this task by clustering features into similarity networks and detecting characteristic neutral losses using predefined equations.^16,17^ However, many adduct pattern–matching methods rely on the assumed presence of [M + H]^+^ or [M–H]^−^ ions, which are not consistently detectable across data sets.^10^ In contrast, Correland can identify pseudomolecular ions even if they were not detected by MS due to the weighted system of equations which improves its robustness and efficiency. Another software tool, CAMERA, reported 90% accuracy under optimized conditions using preselected compounds spiked into *Arabidopsis* extracts,^14^ but performance dropped to 32% in more complex data sets, compared to 64% for CliqueMS.^16^ Correland was evaluated under conditions closely reflecting real LC–MS data using manual annotation of the 50 most abundant pseudomolecular ions per ionization mode (details in Supporting Information). Under these conditions, Correland achieved consistent identification accuracies of 86% and 90% in positive and negative ion modes, respectively. These results were further complemented by an evaluation using a mixture of 33 authentic standards, where the identification accuracies were 68% and 94% in positive and negative ion modes, respectively (Table S7). The lower accuracy in positive mode is attributable to the poor ionization of selected compounds in positive mode, making pseudomolecular ion assignment extremely challenging.

## Conclusions

This work presents a standalone software tool for the rapid construction and visualization of metabolite correlation networks directly from LC–MS feature tables. The integrated workflow combines correlation analysis, network construction, pseudomolecular ion identification, and visualization in a single step, without the need for extensive preprocessing or external platforms. Compared to traditional multivariate methods such as Principal Component Analysis (PCA) or Hierarchical Cluster Analysis (HCA), Correland provides explicit representation of direct pairwise associations, improving biological interpretability. By offering a database-independent solution that requires only minimal input data and no specialized computational expertise, Correland lowers the barrier to network-based metabolomics analysis and broadens accessibility across laboratories. The generation of clean, interpretable correlation networks supports transparent hypothesis development, reproducible data interpretation, and network-driven experimental design. Collectively, these features position Correland as a practical and scalable tool with the potential to standardize correlation-based workflows within the metabolomics community.

## Supplementary Material



## Data Availability

The source code and installation package with preprocessed data sets used in this study are available at https://www.prf.upol.cz/keb/correland/. This website also provides a video tutorial for installation guidance and detailed instructions for use. No external or secondary data sets were used in this study.

## Notes

Extended details on the methodology and results; figures illustrating data processing (software screenshots); tables summarizing the validation results of pseudomolecular ion identification; and application to other data (PDF)
