# Supplementary material for: CorrelandSW: Correlation Networks from LC–MS Data
Source: J Am Soc Mass Spectrom. 2026 Apr 22;37(5):1295–302. doi: 10.1021/jasms.6c00065 (PMC13154350; doi:10.1021/jasms.6c00065)
Supplement: Supplementary file 1 [file js6c00065_si_001.pdf]

Supporting Information

Correland^{SW}: Correlation networks from LC—MS data

Andrea Kosinová, Jiří Grúz*

Department of Experimental Biology, Palacký University, Šlechtitelů 27, CZ-783 71, Olomouc, Czech Republic

* jiri.gruz@upol.cz

Content

1 Installation....2

2 Data and algorithms....3

2.1 Data structure....3

2.2 Data filtering....4

2.3 Ion grouping....5

2.3.1 Cliques....5

2.3.2 Pseudomolecular ion identification....5

2.4 Correlation network....9

2.4.1 Recalculation of PCC....10

2. 4.2 Color customisation....10

2.5 Export....11

3 Results of pseudomolecular ion identification....12

4 Application to other data....15

1 Installation

Correland is a standalone software application developed using MATLAB. The standalone version of Correland is compiled with the MATLAB Compiler, allowing operation independently of a MATLAB installation. Administrative privileges are required for successful installation. The software is compatible with computers running the Windows operating system. Two installation methods are available.

1) Systems with MATLAB installed

In this case, only the MATLAB Runtime (version R2019a for Windows) is required. The installation files are available from the MathWorks website:
http://www.mathworks.com/products/compiler/mcr/index.html
The directory containing the Correland program files can be downloaded from https://www.prf.upol.cz/keb/correland/. These files comprise several *.mat files, Excel files, and associated images. The application is executed directly via Correland.exe.

2) **Systems without MATLAB installed**

A standalone installation package can be obtained from https://www.prf.upol.cz/keb/correland/. Following the standard installation procedure (MyAppInstaller_web.exe), the Correland software is installed in the Program Files directory. The application is executed via Correland.exe (Program Files\Correland\application\Correland.exe).
To ensure full functionality, Correland.exe must be executed with administrative privileges. It should be noted that the shortcut generated during installation may not function as intended. In such cases, the installation path (i.e. *Program Files\Correland\application*) must be manually specified in the “Start in” field of the shortcut properties. Further details are provided in the tutorial available on the project website.

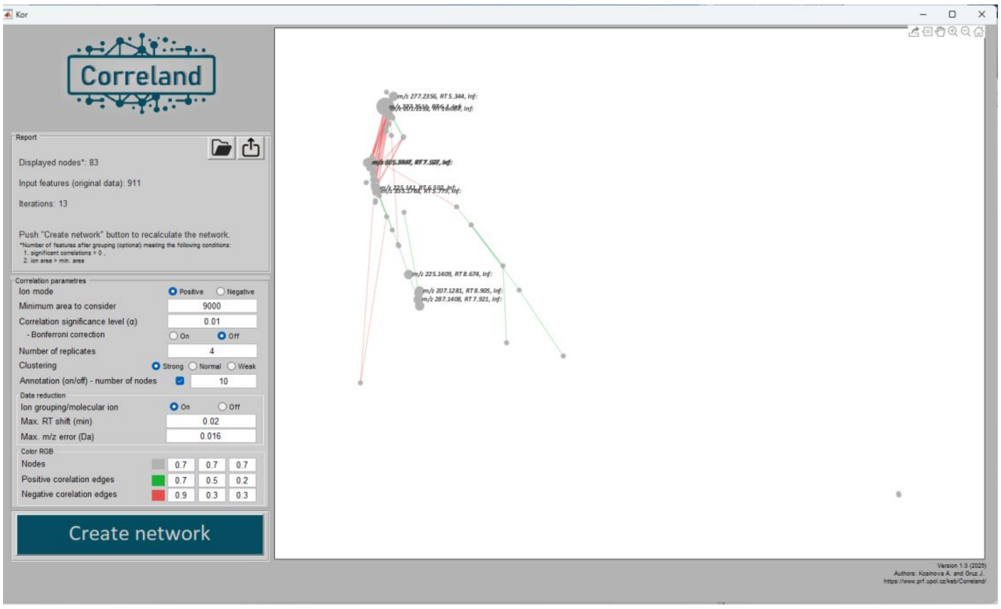

Figure S1. Graphical user interface of the Correland software.

2 Data and algorithms

The primary focus of Correland is the construction of correlation networks from large metabolomic data obtained by liquid chromatography-mass spectrometry (LC–MS). The subsequent paragraphs provide the supporting information for user settings. A simplified scheme of the software is shown in the article (Figure S1).

2.1 Data structure

Coreland is capable of importing data from Excel files. The filled columns must contain the following data: the mass-to-charge ratio (*m/z*), retention time (RT) and peak areas for each sample. This information is referred to as a 'metabolite feature' (one row corresponds to one feature). The annotation of selected ions can be entered in a designated annotation column (Table S1, "Text" column), although it is not mandatory to complete this column. The purpose of the annotation is to define those ions of particular significance, and this is always included in the graph. If the user wishes to identify a number of specific ions, it is possible to set the number to display the most abundant ions, along with their retention time and *m/z* value. If "ion grouping" is allowed, the mass-to-charge ratio refers to the (de-)protonated molecule (pseudomolecular ion).

Each feature must be unique, i.e. different *m/z* and retention time. The automatic control mechanism was developed to prevent duplications in the loaded data. In the event that a data file contains features with identical information across identical columns, the feature is processed on a single occasion. For features sharing the same *m/z* and retention time but differing in integrated area, the feature with the highest total area across the dataset is retained for further analysis. However, both cannot be included in the calculation, as the nodes must be distinct for spatial representation. For general datasets, an annotation column and the corresponding values (in place of ‘area’) for calculation of the correlation matrix are required. The annotation column replaces the *m/z* and RT information and must contain unique identifiers.

Table S1. Example of an Excel file for data entry. Specific ions/features can be annotated in the first column;

Text	*m/z*	RT	Ctrol MS fungi 1	Ctrol MS fungi 2	Ctrol MS fungi 3	Ctrol MS fungi 4	A. Alternata 1	A. Alternata 2	A. Alternata 3	A. Alternata 4	F. Oxysporum 1	F. Oxysporum 2	F. Oxysporum 3	F. Oxysporum 4
	104,142	1,528	3975	3241	2945	2627	4232	3902	3763	3744	4540	4589	3580	3517
feature name	173,1929	1,528	62	35	36	20	2375	1077	1551	2234	8123	6068	4215	5895
	308,1851	1,528	7907	6682	4598	6255	3729	718	2599	3955	3479	2536	2411	2231
	398,1708	1,543	3053	1451	1252	2167	820	354	484	833	581	298	19	69
	212,9086	1,573	2818	3166	2871	2727	1135	1665	902	1049	857	793	957	710
	136,1064	1,576	3701	3177	2755	1723	6424	7052	6222	9178	6314	8789	7961	6312
	514,06	1,717	6934	66	36	72	1877	7	1927	2239	1444	1053	1729	1971
	397,1758	1,729	1993	1541	1311	1709	695	381	498	775	400	350	249	404
	396,1722	1,732	16235	13072	11186	277	5164	2769	3618	5844	2679	2396	143	107

however, this column is optional.

Upon selection of an Excel file for import, the available data selection options are automatically displayed (see Figure S2).

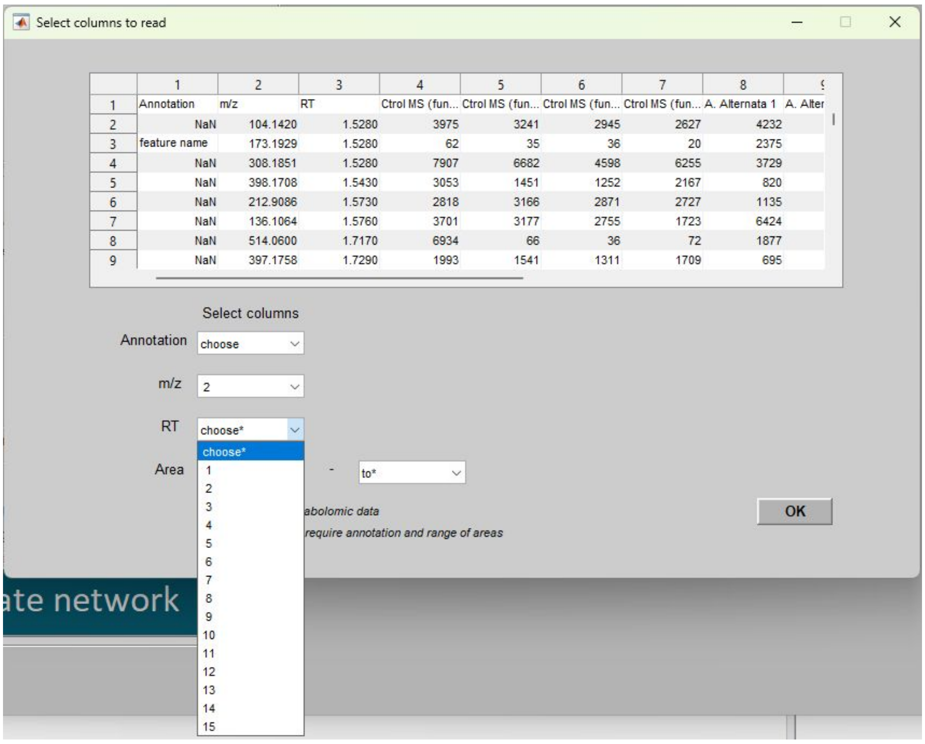

Figure S2. A pop-up window for selecting the columns to be read. The displayed table shows information from the selected Excel file. The user must select the required (*) columns containing the data necessary for calculating the correlation network.

2.2 Data filtering

The software allows the user to configure each parameter in order to extract the most relevant information from the data set. The number of input features incorporated into the correlation network calculation is influenced by two key parameters during the filtering process: the 'Minimum area to consider' and the 'Number of replicates' (see Figure S3). The feature area must be higher than (or equal to) the given threshold 'Minimum area to consider' in at least N samples, where N is given by "'Number of replicates'. Each metabolite feature (row) in the Excel file used for subsequent calculations must comply with the thresholds. This limitation results in the filtration of non-essential information (noise) from the data set.

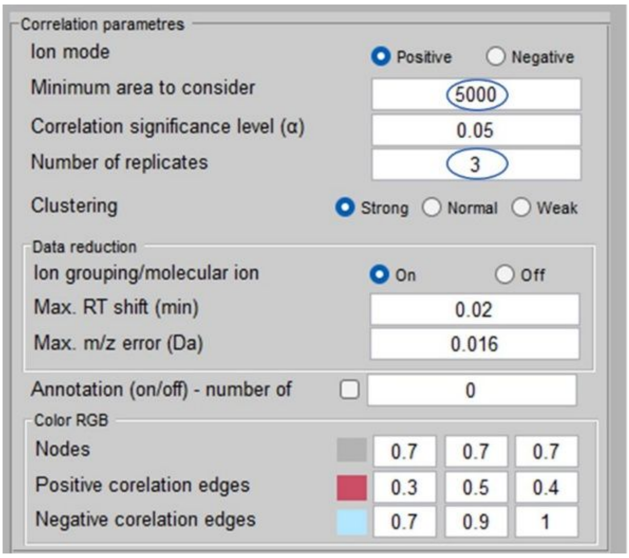

Figure S3. The blue circle highlits the parameters applied during the filtering process.

The process of setting the thresholds for 'Minimum area to consider '(set to 5000; Figure S3) and 'Number of replicates' (set to 3; Figure S3) is illustrated using an example of a feature that meets the specified limits (see Table S2). The sorting is performed automatically, and the order of samples exceeding the threshold is inconsequential.

Table S2. The example of feature meets the thresholds: 'Minimum area to consider'≥5000, 'Number of replicates'≥3. Values that meet the defined thresholds are highlated in green. As three values fulfill these criteria, the feature is included in the subsequent calculation.

1	Text	m/z	RT	Area	area	area	area	area	area	area	area	area
234		115,0038	1,867	7654,876	1097,843	4169,973	8619,114	1830,795	5262,627	4753,635	922,5838	4528,066

2.3 Ion grouping

Grouping of features is essential for simplifying dense networks and is particularly recommended for large metabolomic datasets. The selected ionisation mode is crucial for the calculation of (de-)protonated molecule (pseudomolecular ions), as different equations are applied depending on the mode. The pseudomolecular ion, represents a group of features (clique) sharing the same retention time.

2.3.1 Cliques

The selection of the features belonging to a single clique depends on the knowledge of the measurement accuracy, which is used to determine the optimal sorting parameter, namely the 'maximum RT shift'. Cliques are formed from features exhibiting similar retention times (as described in the manuscript). Subsequently, the features within each clique are validated by calculating the relative standard deviation (RSD). The ratio of the areas of features within a single clique to that of the base peak should be consistent, as these ions represent different forms of the same metabolite (e.g., isotopes, adducts, or fragments). Consequently, they are expected to share the same retention time and chromatographic profile under consistent experimental conditions. If a feature exceeds the RSD limit (set at 25%), it is considered outside the clique and may be reassigned. This threshold was experimentally optimized for metabolomic data. To avoid bias in the resulting RSDs, outliers are removed and RSDs are recalculated. Specifically, one-third of the values furthest from the mean are sequentially eliminated. This procedure ensures that only features that are truly distinct are reorganised.

2.3.2 Pseudomolecular ion identification

The pseudomolecular ion is identified by a system of equations defining typical fragments and adducts under specific experimental conditions. These equations describe the relationships between ions and are used to determine the identity of the pseudomolecular ion. The significance (weight w_r) of each equation is incorporated into the algorithm and can be modified by the user in the Excel file that the software utilises (see Table S3). Consequently, it is possible to incorporate additional equations into the algorithm and assign weights to these equations for inclusion in the calculation. The system of equations proposed for the positive mode has the same form, made up of different equations, described in the manuscript (see Figure 2, paragraph "Psedomolecular ion identification").

Table S3. The clipping of the Excel file, which contains a list of adducts and fragments that together create the system of equations for pseudomolecular ion calculation in negative ion mode. This list can be modified as required by the user.

equation1		equation2		differences	weight
M-H	-1,00783	M-(2*H+O)-H	-19,01839	18,010565	0
M-H	-1,00783	M+Na-(2*H)	20,97412	-21,981945	0
M-H	-1,00783	M+Cl	34,968853	-35,976678	2
M-H	-1,00783	M+K-(2*H)	36,948058	-37,955883	0
M-H	-1,00783	2M-H	-1,007825	0	10
M-H	-1,00783	M+(2*H+C+2*O)-H	44,997655	-46,00548	0
M-H	-1,00783	2M+Na-2H	20,97412	-21,981945	0
M-H	-1,00783	M+(2*C+4*H+2*O)-H	59,013305	-60,02113	0
M-(2*H+O)-H	-19,0184	M-H	-1,007825	-18,010565	0
M-(2*H+O)-H	-19,0184	M+Na-(2*H)	20,97412	-39,99251	0
M-(2*H+O)-H	-19,0184	M+Cl	34,968853	-53,987243	0
M-(2*H+O)-H	-19,0184	M+K-(2*H)	36,948058	-55,966448	0
M-(2*H+O)-H	-19,0184	M+(2*H+C+2*O)-H	44,997655	-64,016045	0
M-(2*H+O)-H	-19,0184	2M+Na-2H	20,97412	-39,99251	0
M-(2*H+O)-H	-19,0184	M+(2*C+4*H+2*O)-H	59,013305	-78,031695	0
M-(2*H+O)-H	-19,0184	2M-H	-1,007825	-18,010565	0

Following the implementation of the filtering process, the features in question are organised in descending order, which also applies to the cliques and ions present in a given clique. The maximum permissible error (namely Max. *m/z* error) e_r for the calculation of pseudomolecular ions can be modified by the user, thus enabling the adaptation of the algorithm to suit the specific data set in question. If no feature belongs to a clique, then the clique consists of the base peak, which will be identified as a pseudomolecular ion. Otherwise, the selection of the pseudomolecular ion is subject to the following steps:

I. Isotopic peak identification

The pairwise differences between the *m/z* values of features within a clique are calculated as

$d_i = m/z_i - m/z_{i+1}$, where $i=1,2,\ldots,n-1$

A potential isotopic peak with m/z_{i+1} is excluded from further consideration if it satisfies both of the following criteria:

1. The mass difference satisfies $|d_i| \leq 1{,}008 + e_r\sqrt{2}$, where e_r represents the mass error tolerance.
2. The maximum measured peak area of m/z_{i+1} is smaller than that of m/z_i.

This approach ensures that only features corresponding to the same metabolite are retained within a clique and effectively removes co-eluting compounds prior to the identification of the pseudomolecular ion. The resulting maximum permissible error (e_r) accounts for the combination of independent and random error sources during the pseudomolecular ion identification process. Assuming that the individual errors are equivalent, their combination is expressed as the square root of the sum of squares, resulting in $e_r\sqrt{2}$.

II. The mass differences matching

For each pair of features originating from a single clique, the algorithm computes the differences between their *m/z* values and searches for identical differences within the system of equations, while considering the maximum permissible error (e_r). The identified feature pairs

and their corresponding equations are then stored, together with their associated weights, in a memory box. The total weight consists of two components: (i)the weight of the pair of equations w_r (as listed in the Excel add-in file), and the (ii)sum of both normalised features areas w_a, calculated relative to the base peak area. As the difference calculation is performed for every possible pair of elements, each equation is detected twice (see Figure S4). Subsequently, the features within the memory box that appear twice are merged, and their weights are aggregated. Consequently, the total weight for a specific equation must exceed a value of 4, even though the corresponding weight in the Excel file is only 2.

m/z₁	*m/z₂*	Equation	w_r	w_a	w_{total}	Pseudomolekular ion *(m/z)*
339.0556	679,1473	$[M-H]^-$ and $[2M-H]^-$	10	0.6+0.5	11.1	$\frac{[m/z_1+H]+[m/z_2+H]}{2}$ - H<br>$\frac{340.0634+340.0776}{2}$-1.007825 = 339.0627
679,1473	339.0556	$[2M-H]^-$ and $[M-H]^-$	10	1.1	11.1	339.0627
…	…	…	…	…	…	…

Pseudomolecular ion *(m/z)*	Total weigh
339.0627	22.2
…	…

Figure S4. Example of calculation in the memory box. The identified equations and corresponding features are used to calculate the pseudomolecular ion.

The highest-scoring combination (w_r + w_a) of feature pairs and the corresponding ions (fragments or adducts) is selected for pseudomolecular ion calculation, with the final value expressed as the average of the calculated pseudomolecular ions (Figure S4, Figure S5). The system of equations can be customized by the user within the Excel add-in file, in accordance with the weight calculation algorithm described above. These configuration files are included in the installation package for both ionisation modes.

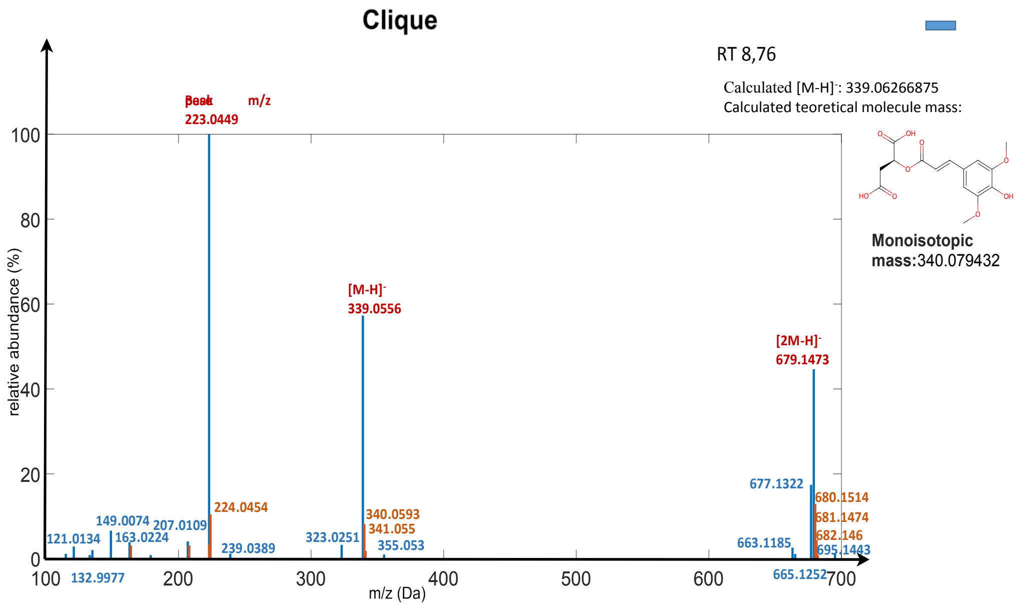

Figure S5. The example of the pseudomolecular ion identification within a single clique. The isotopic peaks not included in the clique are colored orange, while the base peak and the combination of equations used for pseudomolecular ion identification are colored red. The information about monoisotopic mass is available on web page: https://www.chemspider.com/Chemical-Structure.10128116.html

The pseudomolecular ion is identified in the first step if the weight exceeds the given limit, which was set to 4 based on the optimisation process (explained above). Otherwise, the calculated molecular ion (candidate #1) is stored in the memory box, if it meets two other pre-defined criteria:

a) The *m/z* value of the pseudomolecular ion, as derived from the equations, must exceed that of the base peak.

b) The *m/z* value of the calculated pseudomolecular ion must be present within the clique.

The list of candidate pseudomolecular ions is updated throughout the subsequent steps of the identification workflow.

III. Neutral loss identification

The algorithm is based on the knowledge of the typical neutral loss, with the base peak frequently being a highly stable fragmentation product, making it of primary importance. Details of this procedure are provided in section Pseudomolecular Ion Identification (III.) of the manuscript.

IV. The identification of a third potential candidate to the pseudomolecular ion

The third pseudomolecular ion candidate is selected from clique features with *m/z* values exceeding that of the base peak and peak areas greater than half the base peak area. The feature with the highest *m/z* among these is chosen (Figure S6). If no feature satisfies these criteria, candidate #3 is assigned as the base peak.

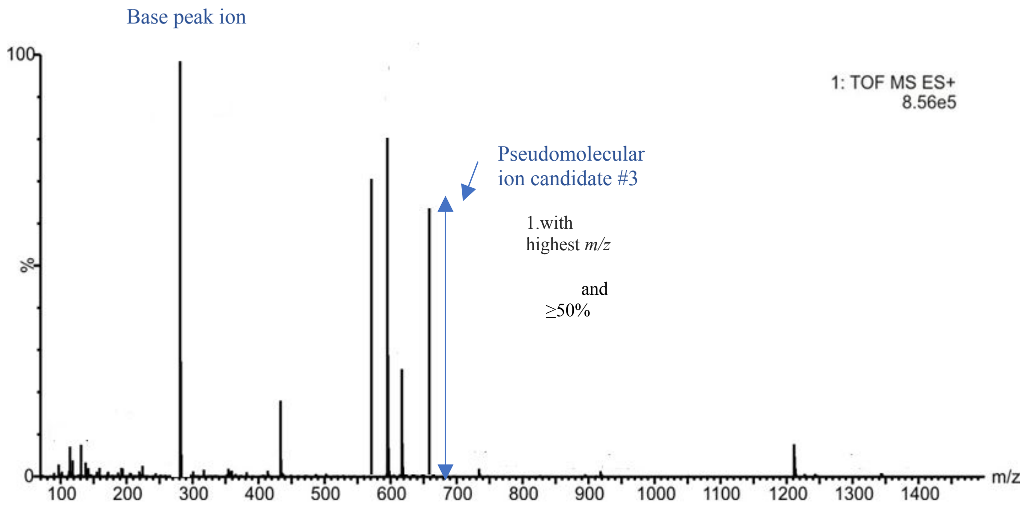

Figure S6. Example of the identification of the third potential pseudomolecular ion candidate – section Pseudomolecular Ion Identification (V.) in the manuscript for details.

If a match is found among the candidate pseudomolecular ions, the corresponding pseudomolecular ion is considered successfully identified. If no match is found, the candidate with the highest *m/z* is selected to represent the group as the pseudomolecular ion. A simplified scheme of pseudomolecular ion identification is given in Figure S7.

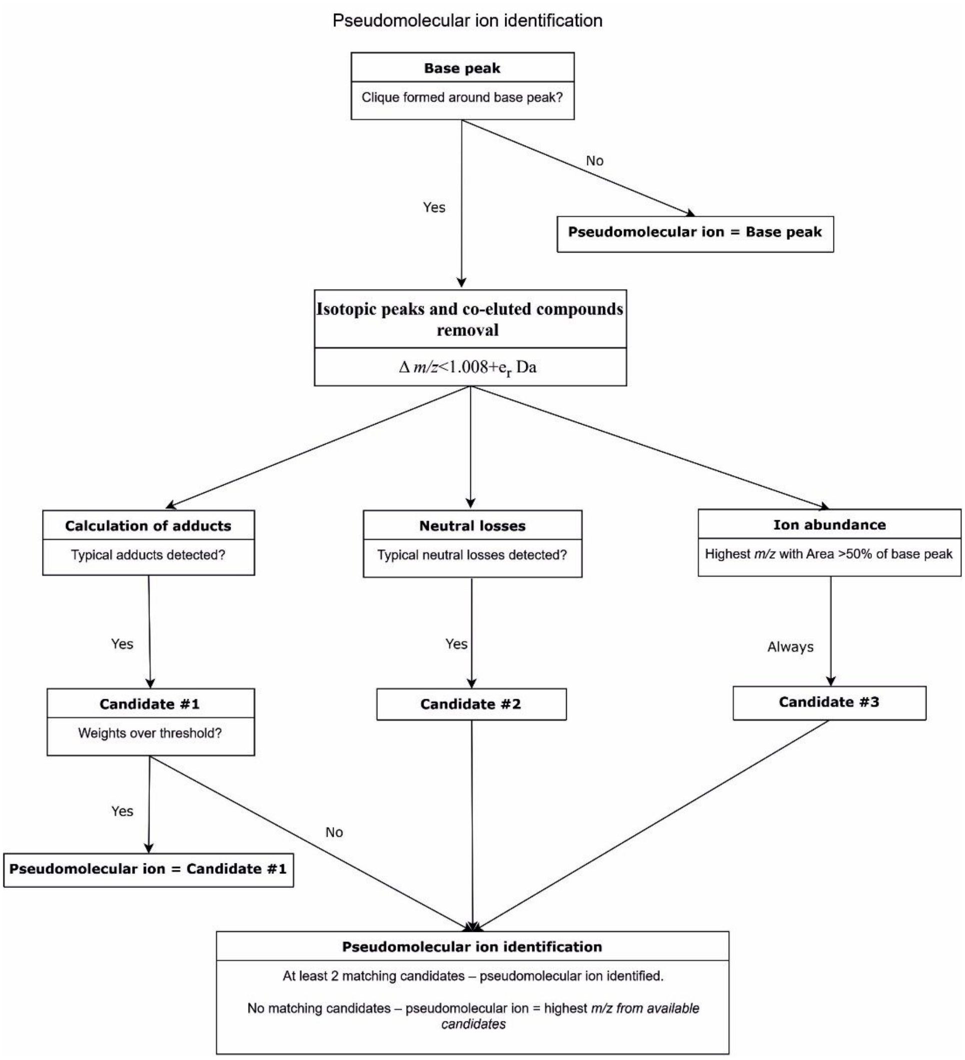

Figure S7. Simplified scheme of the process of pseudomolecular ion identification

2.4 Correlation network

The generation of the correlation network is dependent on the calculation of a correlation matrix. Within this matrix, the Pearson correlation coefficient r and the p-value are calculated for each pair of features/cliques, based on the feature area:

$$r = \frac{\sum_{i=1}^{n} X_i y_i - n\bar{x}\bar{y}}{(\mathrm{n}-1) s_x s_y},$$

where x_i and y_i represent the areas of features (nodes) between which we are seeking a relationship, n denotes the number of areas to be consider and s_x and s_y are the standard deviations of parameters x and y.

The parameter α can be configured by the user. The correlation network will then only be generated from correlations, that meet the specified threshold. The correlation value will only be used for the network calculation if it is strong, as specified $p < \alpha$, where a significance level of α=0.05 or α=0.01 is recommended. A less stringent value may have a detrimental effect on the ability to identify relevant relationships between ions. Furthermore, the number of end nodes is also affected by the stringency of the α parameter. A Bonferroni correction may be applied to adjust the significance threshold (α) for multiple comparisons.

2.4.1 Recalculation of PCC

The recalculation of the Pearson correlation coefficient r provides a more effective visualisation of the nodes in space (spatialisation) by enabling a more precise distinction between positive and negative correlations (edges). The clustering efficiency can be set as strong, normal or weak. In order to differentiate between high negative correlations, it is recommended that the strong level be used for features that are highly correlated.
The recalculation for positive correlations $r > 0$ is defined as follows:

$$s(1 - |r^x|),$$

The weight calculation for negative correlations ($r \leq 0$) is defined as follows:

$$s + |r^x|(1 - s),$$

where $s = 0.1$ and $x = 2$ for both equations have been optimised to ensure a clean graphical output for strong clustering. The recalculation process generates the weights approaching 0 for a strongly positive correlation ($r \sim 1$). Conversely, a strongly negative correlation ($r \sim -1$) results in a weight approaching 1. This indicates that negatively correlated nodes are displaced from each other, while positively correlated nodes are attracted to each other. The parameters $s = 0.3$ and $x = 8$ were determined for normal clustering, while $s = 0.5$ and $x = 16$ were identified for weak clustering. The recalculation of the weights is shown in the table below (see Table S4).

Table S4. The table presents the results of the recalculation of the highly correlated features for spatialisation, where the higher weights produce longer edges.

PCC/Clustering	strong	normal	weak
⟨-0.9; -0.99⟩	⟨0.829; 0.982⟩	⟨0.6013; 0.946⟩	⟨0.593; 0.926⟩
⟨0.9; 0.99⟩	⟨0.019; 0.00199⟩	⟨0.170; 0.0232⟩	⟨0.407; 0.074⟩

After rescaling, edge reduction is used to improve visual clarity and to minimise the number of intersecting edges, depending on the number of nodes displayed. The article provides comprehensive details on the process.

2. 4.2 Color customisation

The customisation of graphic output is achievable through the configuration of edge or node colors (Figure S8). The underlying foundation of this customisation is the RGB system, which operates within the numerical range of ⟨0;1⟩. In this system, the color black is represented by the setting R=0; G=0; B=0, and white by the setting R=1; G=1; B=1. Once the number in a given cell has been modified and the other cell pressed, the resulting color is projected onto the square field in close proximity.

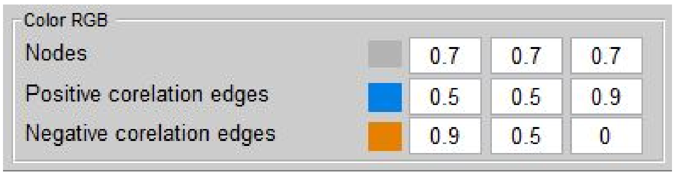

Figure S8. The option to optimise the coloring of nodes and edges according to the user's preferences.

2.5 Export

A variety of export options are available to users. Graphical output can be saved as an image in a range of formats, including image, document or metafile (.svg, .png, .pdf and .tif). Information pertaining to the end nodes, weights (recalculated *r*) and correlation coefficients can be exported as an Excel file.

Two distinct buttons facilitate data export. One of these buttons is a specialised button for saving images as metafile, or for the exporting of information about end nodes. This button is accessible from the main menu (see Figure S9).

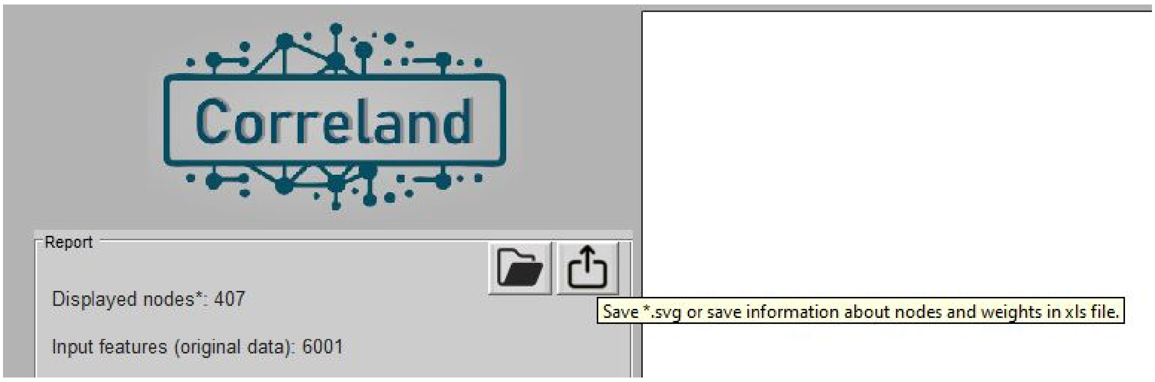

Figure S9. The facility to export data in the SVG and XLS formats is accessible via the main menu.

The second button is accessible directly from the output graph (upper right corner), which is a MATLAB-incorporated function. This button allows the user to save the generated image in a variety of formats, including Portable Document Format (PDF) (see Figure S10).

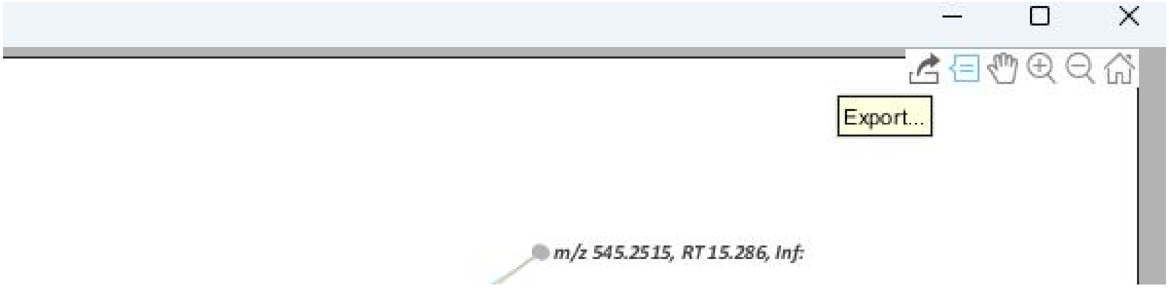

Figure S10. The button enables the export of data in a variety of image and PDF formats.

3 Results of pseudomolecular ion identification

Table S5. The results of the pseudomolecular ion identification testing, conducted on data derived from an LC-MS analysis of model plant *Arabidopsis thaliana*. measured in negative ion mode; Used parameters: 'Minimum area to consider' = 300; 'Correlation signification level (α)' =0.05; 'Number of replicates' = 3; 'Max. RT shift (min)'= 0.02; 'Max. *m/z* error (Da)'= 0.016.

Base peak (negative mode)	MSI identification level[a]	Size of clique	Calculated *m/z* of $[M-H]^-$	Correct?
RT 6.514; *m/z* 385.0976	2 (Sinapic acid glucoside)	45	385.1059	OK
RT 8.767; *m/z* 223.0449	1 (Sinapoyl malate)	38	339.0627	OK
RT 10.852; *m/z* 591.1679	2 (Sinapoyl glucose 1)	15	591.1736	OK
RT 8.845; *m/z* 577.1487	2 (Kaempferol-Rha-Rha)	29	577.1558	OK
RT 5.167; *m/z* 387.113		13	341.1111	OK
RT 7.168; *m/z* 609.1387	2 (Quercetin-Rha-Glu)	26	609.1387	OK
RT 8.014; *m/z* 593.1466	2 (Kampferol-Rha-Glu)	16	593.154	OK
RT 15.286; *m/z* 545.2515	2 (dinor-OPDA monogalactosyl monoacylglycerol)	22	499.2479	OK
RT 8.812; *m/z* 917.2458	4	21	917.2458	WRONG (heterodimer 349 + 577)
RT 9.337; *m/z* 463.1354	4	9	417.1286	OK
RT 7.924; *m/z* 593.1466	2 (Kaempferol-Rha-Glu 2)	27	593.1547	OK
RT 8.947; *m/z* 223.0449	1 (Sinapic acid)	9	223.0449	OK
RT 16.069; *m/z* 735.3455	4	18	689.3446	OK
RT 18.841; *m/z* 531.2699	4	15	485.2703	OK
RT 11.794; *m/z* 591.1679	2 (Disinapoyl glucose 2)	7	591.1755	OK
RT 8.989; *m/z* 339.0556	2 (Sinapoyl malate isomer)	17	339.0627	OK
RT 7.363; *m/z* 643.2398	4	10	643.2398	WRONG (dimer of 321)
RT 13.063; *m/z* 431.0863	4	5	431.093	OK
RT 5.542; *m/z* 417.1255	4	8	371.1202	OK
RT 8.11; *m/z* 623.1572	4	15	623.1657	OK
RT 7.384; *m/z* 307.0705	2 (9,10-dihydro-hydroxyjasmonate sulphonate)	1	307.0705	OK
RT 6.025; *m/z* 755.2042	2 (Quercetin-Rha-Glu-Rha)	15	755.2048	OK
RT 12.973; *m/z* 789.2614	4	10	789.2626	OK
RT 5.791; *m/z* 399.0785	2 (Scopolin)	6	399.0785	WRONG
RT 7.582; *m/z* 825.2486	4	7	825.2486	OK
RT 4.615; *m/z* 285.0447	3 (Gentisic acid derivative)	7	285.0505	OK
RT 16.879; *m/z* 573.2835	2 (OPDA monogalactosyl monoacylglycerol)	7	527.2774	OK
RT 4.588; *m/z* 323.1178	4	6	323.1245	OK
RT 13.975; *m/z* 707.3094	4	6	661.3055	OK
RT 17.548; *m/z* 459.2045	4	2	459.2045	OK
RT 18.058; *m/z* 263.1484	2 (dinor-OPDA)	2	263.1484	OK
RT 9.982; *m/z* 579.2013	4	6	579.2013	OK
RT 17.827; *m/z* 693.3329	4	11	647.3255	OK
RT 10.789; *m/z* 398.0949	4	9	352.0882	OK
RT 8.134; *m/z* 423.1734	4	9	467.1728	OK

RT 13.315; *m/z* 591.1675	3 (Sinapic acid derivative)	2	591.1675	OK
RT 11.08; *m/z* 725.2078	3 (Scopoletin derivative)	3	725.2078	OK
RT 12.934; *m/z* 476.0963	2 (8-methylthiooctyl glucosinolate)	1	476.0963	OK
RT 5.989; *m/z* 227.1208		2	227.1208	OK
RT 13.093; *m/z* 789.2614		9	789.2626	OK
RT 5.575; *m/z* 385.0974	2 (sinapic acid glucosylester)	5	385.1045	OK
RT 2.305; *m/z* 297.1012	4	4	297.1012	OK
RT 3.628; *m/z* 587.177	4	8	293.0769	OK
RT 5.701; *m/z* 492.0944	2 (glucohirsutin)	4	492.0944	OK
RT 9.565; *m/z* 687.164	4	9	687.164	OK
RT 7.876; *m/z* 1341.3768	4	14	1359.3925	WRONG
RT 8.899; *m/z* 435.1225	4	10	435.1292	OK
RT 4.474; *m/z* 549.1743	4	8	549.1743	WRONG
RT 7.288; *m/z* 352.0889	4	2	352.0889	OK
RT 16.741; *m/z* 927.3753	4	8	927.3753	OK

[a]1 – authentic standard, 2 – putative identification, 3 – putative compound class, 4 – unknown

Table S6. The results of the pseudomolecular ion identification testing, conducted on data derived from an LC-MS analysis of model plant *Arabidopsis thaliana* in positive ion mode; Used parameters: 'Minimum area to consider' = 300; 'Correlation signification level (α)' =0.05; 'Number of replicates' = 3; 'Max. RT shift (min)' = 0.02; 'Max. *m/z* error (Da)'= 0.016.

**Base peak (positive mode)**	**MSI identification level[a]**	**Size of clique**	**Calculated *m/z* of pseudomolecular ion $[M+H]^+$**	**Correct?**
RT 11.209; *m/z* 202.1409	2 (8-MeSO-Octyl-CN)	13	202.1424	OK
RT 17.107; *m/z* 234.1158	2 (8-MeSO-Octyl-NCS)	24	234.1196	OK
RT 8.815; *m/z* 287.0775	4	12	579.2325	OK
RT 8.722; *m/z* 207.0788	4	30	125.0677	WRONG
RT 6.502; *m/z* 207.0787	4	25	225.0892	OK
RT 10.843; *m/z* 575.2363	4	24	593.2496	OK
RT 4.174; *m/z* 192.1541	4	4	192.153	OK
RT 8.746; *m/z* 188.1215	2 (7-MeSO-Heptyl-CN)	6	188.1228	OK
RT 7.162; *m/z* 611.2259	2 (Quercetin-Rha-Glu)	12	611.2268	OK
RT 5.83; *m/z* 277.1889	2 (Coumaroylagmatine)	10	277.1883	OK
RT 15.922; *m/z* 220.0996	4	6	220.1007	OK
RT 15.283; *m/z* 321.2342	4	29	501.3221	OK
RT 5.776; *m/z* 193.0622	2 (scopolin)	5	355.1357	OK
RT 7.876; *m/z* 1343.5277	4	6	1343.5277	OK
RT 5.983; *m/z* 229.171	4	5	229.1691	OK
RT 17.56; *m/z* 318.3274	4	4	318.3274	OK
RT 17.485; *m/z* 274.2962	4	3	274.2962	OK
RT 8.008; *m/z* 595.2277	2 (Kampferol-Rha-Glu)	8	595.2277	OK
RT 4.645; *m/z* 188.082	4	12	219.0998	WRONG
RT 2.977; *m/z* 146.0717	4	13	146.0706	WRONG
RT 3.199; *m/z* 120.0864	1 (Phenylalanine)	7	166.0945	OK
RT 3.577; *m/z* 240.0683	4	3	240.0683	OK
RT 2.023; *m/z* 268.1245	1 (Adenosine)	5	268.1245	OK
RT 18.271; *m/z* 230.2643	4	2	230.2643	OK
RT 16.066; *m/z* 349.2691	4	23	691.4327	OK
RT 8.101; *m/z* 625.2475	4	9	625.2475	OK

RT 9.964; *m/z* 203.1254	4	11	221.138	OK
RT 9.325; *m/z* 436.2247	4	26	419.1975	OK
RT 13.057; *m/z* 287.0773	4	4	433.1556	OK
RT 17.95; *m/z* 288.3124	4	2	288.3124	OK
RT 11.281; *m/z* 176.1576	4	4	176.1566	OK
RT 12.499; *m/z* 336.1363	3 (Indole derivative)	7	336.1385	OK
RT 5.53; *m/z* 193.0985	4	15	373.1853	OK
RT 18.841; *m/z* 325.2663	4	27	487.3416	OK
RT 17.407; *m/z* 187.0974	4	11	133.0972	WRONG
RT 4.594; *m/z* 160.0884	4	10	160.0889	OK
RT 11.791; *m/z* 369.1518	3 (Sinapic acid derivative)	9	185.0882	WRONG
RT 16.204; *m/z* 532.3565	4	4	532.3565	OK
RT 8.77; *m/z* 130.0711	4	3	130.0711	WRONG
RT 6.01; *m/z* 303.0767	4	6	757.3047	OK
RT 5.149; *m/z* 163.0854	4	16	343.174	OK
RT 16.879; *m/z* 321.2342	4	21	339.2476	OK
RT 2.752; *m/z* 124.0439	4	2	286.1141	OK
RT 6.442; *m/z* 174.1047	4	4	174.1062	OK
RT 8.872; *m/z* 193.062	4	7	437.1935	OK
RT 18.613; *m/z* 349.2693	4	11	367.2821	OK
RT 17.596; *m/z* 160.085	4	4	160.085	OK
RT 14.767; *m/z* 163.0487	4	3	163.0487	OK
RT 18.19; *m/z* 172.1791	4	2	172.1791	OK
RT 18.058; *m/z* 265.2	4	4	265.1985	OK
RT 16.681; *m/z* 482.2714	4	16	465.2451	OK

[a]1 – authentic standard, 2 – putative identification, 3 – putative compound class, 4 – unknown

Table S7. The results of the pseudomolecular ion identification by Correland performed on the mixture of standards. Applied parameters: 'Minimum area to consider' = 1000; 'Correlation signification level (α)' =0.05; 'Number of replicates' = 0; 'Max. RT shift (min)' = 0.01; 'Max. *m/z* error (Da)'= 0.01.

Standard	RT	*m/z* neg	Correct?	*m/z* poz	Correct?
4-hydroxybenzoic acid	6.55	137	WRONG	139	WRONG
3-hydroxybenzoic acid	6.99	137	OK	139	WRONG
salicylic acid	10.49	137	OK	139	WRONG
D4-4-hydroxybenzoic acid	5.73	141	OK	143	OK
D4-salicylic acid	10.46	141	OK	143	WRONG
trans-cinnamic acid	14.18	147	OK	149	OK
3,5-dihydroxybenzoic acid	4.36	153	OK	155	WRONG
protocatechic acid	4.10	153	OK	155	WRONG
gentisic acid	5.31	153	OK	155	WRONG
4-coumaric acid	8.25	163	OK	165	OK
3-coumaric acid	8.94	163	OK	165	WRONG
2-coumaric acid	10.89	163	OK	165	OK
vanillic acid	6.49	167	OK	169	OK
gallic acid	2.58	169	OK	171	OK
caffeic acid	6.53	179	OK	181	OK
ferulic acid	8.99	193	OK	195	OK
isoferulic acid	9.47	193	OK	195	OK
syringic acid	6.74	197	OK	199	OK
sinapic acid	9.55	223	OK	225	OK
chrysin	17.72	253	OK	255	OK
apigenin	15.03	269	OK	271	OK
genistein	18.00	269	OK	271	OK
naringenin	15.06	271	OK	273	OK
pinobaksin	15.37	271	OK	273	OK
kaempferol	15.32	285	OK	287	OK
eriodictyol	13.07	287	OK	289	OK
catechin	6.55	289	OK	291	WRONG
quercetin	13.27	301	OK	303	OK
hesperetin	15.60	301	OK	303	OK

taxifolin	9.38	303	OK	305	WRONG
rhamnetin	16.84	315	OK	317	OK
chlorogenic acid	5.53	353	OK	355	OK
rutin	8.54	609	OK	611	OK
			**Success rate 94.1%**		**Success rate 67.6%**

4 Application to other data

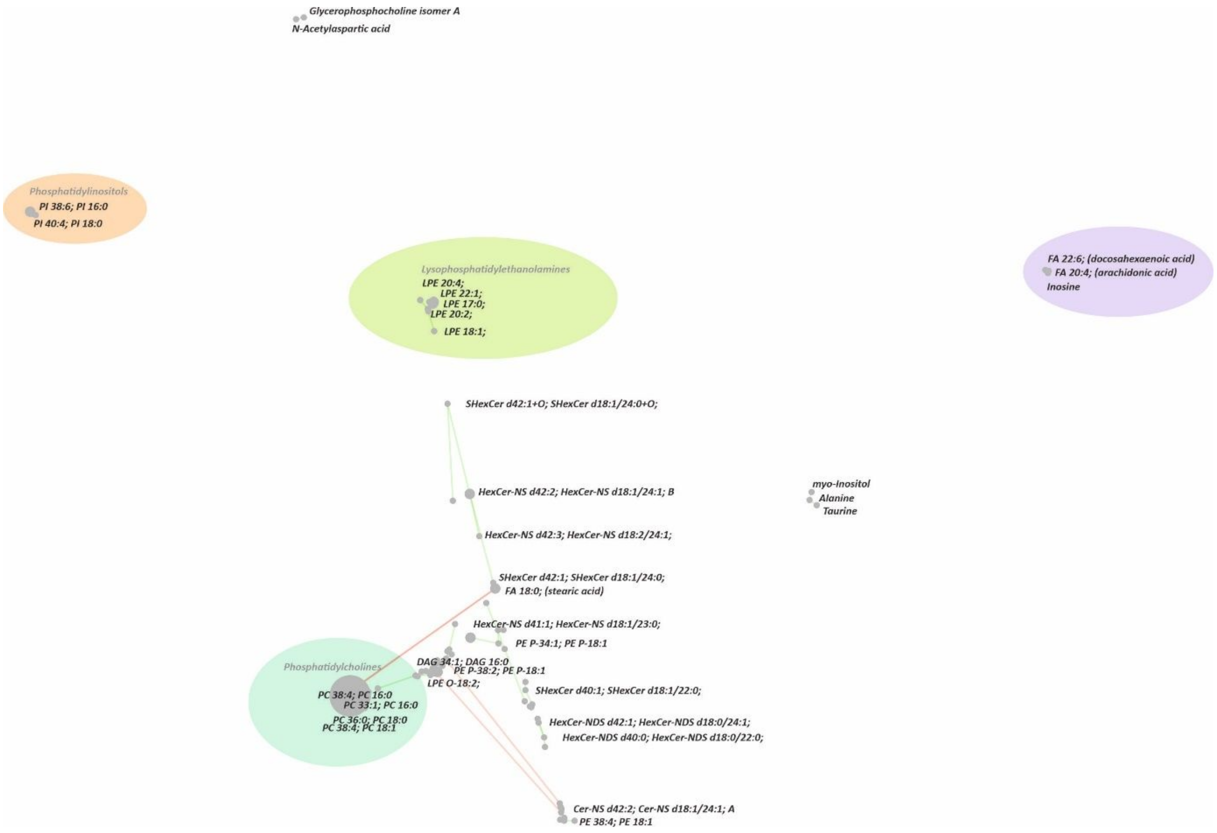

Figure S11. The resulting correlation network and spatialization of metabolites after aplication of Correland tool to the annotated metabolomic data of an aging (3-92 weeks) mouse brain – pons (Ding et al., 2021). All details about this study including raw data are available at https://pmc.ncbi.nlm.nih.gov/articles/PMC8519999/. Applied parameters: Minimum area to consider: 50000; Number of replicates: 8; Correlation significance level (α): 0.01 (adjusted); Bonferroni correction: On; Clustering: Strong; Ion grouping: Off. The analysed metabolites in the referenced study were annotated and thus, no ion grouping was applied. To reduce the number of dispayed nodes, minimum area of 50000 and Bonferroni correction were applied. Group highlighting (elipses) was applied following export from the Correland software. The labels (annotations) were manually moved/removed to improve readability.
